# Postoperative Use of the Chemopreventive Vitamin K2 Analog in Patients with Hepatocellular Carcinoma

**DOI:** 10.1371/journal.pone.0058082

**Published:** 2013-03-07

**Authors:** Jian-Hong Zhong, Xin-Shao Mo, Bang-De Xiang, Wei-Ping Yuan, Jin-Fang Jiang, Gui-Sheng Xie, Le-Qun Li

**Affiliations:** 1 Hepatobiliary Surgery Department, Tumor Hospital of Guangxi Medical University, Nanning, People's Republic of China; 2 Chemotherapy Department, Tumor Hospital of Guangxi Medical University, Nanning, People's Republic of China; 3 General Surgery Department, The Third Affiliated Hospital of Guangxi Medical University, Nanning, People's Republic of China; Yonsei University College of Medicine, Korea, Republic of

## Abstract

**Aim:**

To evaluate the chemopreventive efficacy of vitamin K2 (VK2) analog in patients with hepatocellular carcinoma (HCC) after curative hepatic resection or local ablation, since a recent randomized control trial (RCT) and systematic review have given contradictory results.

**Methods:**

MEDLINE, EMBASE and Cochrane library databases were systematically searched through the end of May 2012. Meta-analysis of RCTs and cohort studies was performed to estimate the effects of the VK2 analog on tumor recurrence rate and overall survival (OS). Risk ratios (RRs) and 95% confidence intervals (95% CIs) were calculated.

**Results:**

Six RCTs and one cohort study involving a total of 930 patients were included. VK2 analog therapy did not reduce the 1-year recurrence rate, with a pooled RR of 0.67 (95% CI 0.39–1.13, p = 0.13). However, VK2 analog therapy was associated with a significant reduction in the 2- and 3-year tumor recurrence rates, with respective pooled RRs of 0.65 (95% CI 0.51–0.83, p<0.001) and 0.70 (95% CI = 0.58–0.85, p<0.001). The therapy was also associated with a significant improvement in 1-, 2-, and 3-year OS, with respective pooled RRs of 1.03 (95% CI 1.01–1.05, p = 0.02), 1.11 (95% CI 1.03–1.19, p = 0.005) and 1.14 (95% CI 1.02–1.28, p = 0.02). None of the studies reported adverse effects attributable to VK2 analog therapy.

**Conclusion:**

The VK2 analog may reduce recurrence rate after 1 year and improve OS in HCC patients as early as 1 year. However, these findings should be considered preliminary since the majority of patients came from an RCT with survival data out to only 1 year. More extensive studies with larger sample sizes and longer follow-up are needed.

## Introduction

As a major public health problem worldwide, hepatocellular carcinoma (HCC) is the third leading cause of cancer-related deaths [1]. Despite advances in our understanding of the biology and natural history of HCC and marked improvements in diagnostic techniques, the prognosis for HCC patients remains discouraging because of the high recurrence rate and frequent incidence of intrahepatic metastasis. HCC patients have a high mortality rate due to high intrahepatic recurrence [2]. As a result, preventing HCC recurrence postoperatively is one of the most important challenges to improving surgical efficacy.

Various forms of postoperative therapies have been reported, such as interferon, transarterial chemoembolization, and adoptive immunotherapy. Adjuvant interferon has a significant beneficial effect after curative surgery for HCC [3]. However, interferon is frequently associated with adverse effects. Postoperative transarterial chemoembolization seems promising only for HCC patients at high risk of recurrence [4]. Adoptive immunotherapy, while associated with lower recurrence after HCC surgery, does not appear to increase overall survival [5]. Therefore, a more effective and safer postoperative therapy is needed.

A vitamin K2 (VK2) analog, marketed under the name menatetrenone, which is already in use as a novel and safe therapy for osteoporosis [6], was shown in 2004 to prevent recurrence of HCC in women with viral cirrhosis [7]. Since then, several clinical studies have investigated the efficacy of postoperative therapy with VK2 analog in HCC patients. A systematic review [8] based on four randomized controlled trials (RCTs) [9]–[12] involving 209 patients showed that the analog significantly improved tumor recurrence-free survival. However, a recent double-blind, randomized, placebo-controlled study involving 548 patients failed to find an association between postoperative use of the VK2 analog and lower HCC recurrence. In addition to these contradictory findings, this recent RCT failed to address the long-term efficacy of the VK2 analog [13].

To help resolve these questions about the efficacy of VK2 analog therapy, we performed a meta-analysis based on the same studies as in the previous systematic review as well as the most recent large-scale RCT, and we focused on not only short-term but also long-term outcomes.

## Patients and Methods

### Identification of trials

The electronic databases of MEDLINE, EMBASE and the Cochrane Library were systematically searched until the end of May 2012. Eligible studies were identified using any of the following key words: hepatocellular carcinoma, hepatic tumor, liver tumor, postoperative, chemopreventive, vitamin, menatetrenone. A manual search of the relevant references and review articles was performed to identify additional relevant studies. RCTs, quasi-randomized studies and cohort studies were included. Studies identified by the search were screened independently by two reviewers. Any disagreements were arbitrated by a third reviewer.

### Types of patients and interventions

Hepatic resection was considered curative only when the histological resection margin was clear. Local ablation was considered curative when the postoperative computed tomography (CT) scan showed no residual tumors at 1 month after therapy. In the treatment arm, HCC patients received curative treatment followed by oral administration of VK2 analog. In the control arm, patients received curative treatment with or without placebo. Patients were excluded if they were receiving warfarin, which may affect VK2 analog metabolism, or if they received VK2 analog preoperatively. Patients were also excluded if they showed obvious tumor invasion into the portal vein or extrahepatic metastasis.

### Types of outcome measures

The primary outcomes evaluated in this meta-analysis were overall survival (OS) and tumor recurrence rate. The secondary outcome was the incidence of adverse events attributable to the use of oral VK2 analog.

### Quality assessment

Two reviewers independently evaluated all the included RCTs in terms of randomization by sequence generation, allocation concealment, blinding of outcome assessors and reporting of intention-to-treat analysis. Trials were considered to be of low quality if they reported none of the items, of moderate quality if they reported on one or two items and of good quality if they reported on three or four. The reporting of this systematic review is in accordance with the QUOROM statement [14]. Quasi-randomized studies and cohort studies were defined to be of low quality.

### Data extraction

Using a standardized form, two reviewers independently extracted data about author details, methodological quality, sample size, patient characteristics, dosage and outcomes. Discrepancies were resolved by consensus.

### Statistical analysis

Risk ratios (RRs) (Mantel-Haenszel) with corresponding 95% confidence intervals (95% CIs) were calculated for dichotomous outcomes. A fixed-effect model and a random-effect model were applied in an ‘intention-to-treat’ analysis, i.e. all patients were evaluated according to their group allocation. Patients whose endpoint was unknown were considered dead or to have suffered tumor recurrence. Heterogeneity was assessed by calculating I^2^. Homogeneity between trials was analyzed using the χ^2^ test, with significance set at *P*>0.1. The point estimate of the RR was considered statistically significant at the *P*<0.05 level if the 95% CI did not include 1. Data from each study were analyzed using the software package RevMan 5. Publication bias was assessed by visual inspection of Begg's funnel plots.

### Subgroup and sensitivity analysis

Subgroup analysis was performed according to VK2 analog doses. Sensitivity analysis excluding cohort studies was also performed.

## Results

### Identification and characteristics of studies

**Figure 1:**
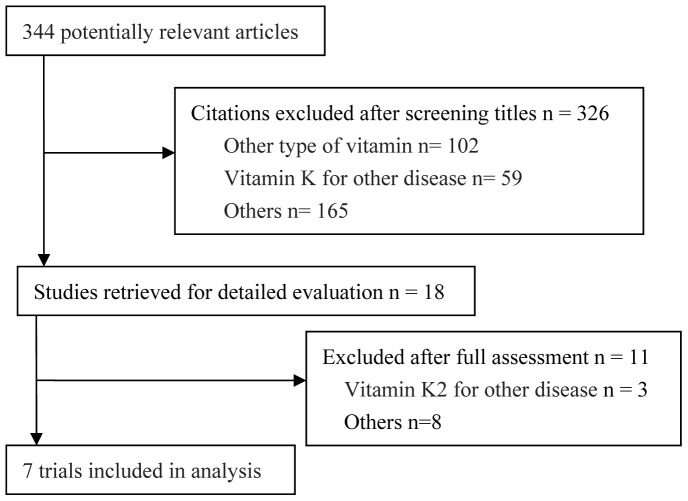
Selection process for trials included in this meta-analysis.

From 344 citations identified by database searches, six eligible RCTs [9]–[13], [15] and one cohort study [16] involving a total of 930 patients were included in this meta-analysis (Fig. 1). All six studies were conducted in Japan. A definite diagnosis of HCC was made based on histological evidence or a combination of several imaging modalities, e.g. hepatic angiography, enhanced CT, and magnetic resonance imaging. All patients with HCC underwent curative surgical resection or local ablation therapy. No patient in either the control or treatment arms received any type of chemopreventive therapy before the discovery of recurrent HCC. Patients in the treatment arm received a daily dose of 45 mg or 90 mg of VK2 analog postoperatively. Only 1-year survival data was extracted from the study by Yoshida H *et al.* [13] The characteristics of the studies included in the meta-analysis are shown in Table 1. After systematically searched the relevant databases again in December 2012, we found one study [15] which published with abstract has full text [17], and regarded as one study. However, no other eligible study was found.

**Table 1:** Characteristics of included studies.

Study	Treatment arm (n)	Age	Gender (M/F)	No. of tumors	Mean tumor size (mm)	Child-Pugh classification (A/B)	No. HBV cases	No. HCV cases	PIVKA-II (mAU/mL)	AFP (ng/mL)
Yoshiji et al. [9]	VK2 (18)	62.8	10/8	1.62	17.9	16/2	0	15	60.2	79.8
	Control (25)	60.5	17/8	1.59	18.7	20/5	3	11	41.3	88.5
Mizuta et al. [10]	VK2 (32)	63.3	23/9	1.50	17.7	26/6	4	29	41.8	102.2
	Control (29)	64.5	18/11	1.48	19.4	22/7	3	27	70.3	508
Kakizaki et al. [11]	VK2 (30)	69.1	17/13	Single: 19 Multiple: 11	20.4	22/8	0	30	≤40: 10 >40: 20	≤20: 15 ≥20: 15
	Control (30)	69.0	18/12	Single: 22 Multiple: 8	25.0	22/8	0	30	≤40: 8 >40: 22	≤20: 16 ≥20: 14
Hotta et al. [12]	VK2 (21)	64.4	16/5	Single: 11 Multiple: 10	11–30: 18 31–50: 3	15/6	6	14	<40: 14 ≥40: 7	<20: 6 ≥20: 15
	Control (24)	69.1	17/7	Single: 9 Multiple: 15	11–30: 18 31–50: 6	12/12	3	19	<40: 9 ≥40: 15	<20: 9 ≥20: 15
Yoshida et al. [13]	VK2 (367)	68.6	234/133	Single: 260 Multiple: 107	20	323/44	38	305	<40: 328 ≥40: 36	<100: 344 ≥100: 22
	Control (181)	68.9	108/73	Single: 127 Multiple: 54	20.3	154/27	20	150	<40: 155 ≥40: 25	<100: 164 ≥100: 17
Kubota et al. [15]	VK2 (50)	65.8	40/10	1.3	13–127	45/5	26	30	50	33
	Control (51)	68.4	38/13	1.3	8–250	46/5	19	28	90	13
Hosho et al. [16]	VK2 (23)	67.0	11/12	1.3	Maximum: 23	-	8	16	1182.9	382.6
	Control (49)	69.0	27/22	1.4	Maximum: 28	-	15	33	2421.3	226.8

Abbreviations: AFP, α-fetoprotein; HBV, hepatitis B virus; HCV, hepatitis C virus; PIVKA-II, prothrombin induced by vitamin K absence or antagonist-II; VK2, vitamin K2 analog.

### Methodological quality of studies

The methodological qualities were good in two studies [13], [15], moderate in two studies [9]–[10] and low in the remaining three [11]–12,16 (Table 2).

**Table 2:** Methodological quality assessment: internal validity of included studies.

Study	Description of random allocation	Concealment of random allocation	Blinding of those assessing treatment effects	Intention-to-treat analysis
Yoshiji et al. [9]	+	+	−	−
Mizuta et al. [10]	+	−	−	+
Kakizaki et al. [11]	−	−	−	−
Hotta et al. [12]	+	−	−	−
Yoshida et al. [13]	+	+	+	+
Kubota et al. [15]	+	+	−	+
Hosho et al. [16]	−	−	−	−

### Therapy outcomes

Outcome data from each study are presented in Table 3. In the control arm, the 3-year recurrence rate ranged from 67.6% to 91.6% and the 3-year OS from 56.5% to 88.0%.

**Table 3:** Treatment outcomes of the included studies.

Study	Postoperative Treatment	Treatment (surgery/local ablation) (n)	Follow-up (mo.)	Recurrence rate (%)			Survival rate (%)		
				1-year	2-year	3-year	1-year	2-year	3-year
Yoshiji et al. [9]	VK2 (45 mg)	0/18	48.0	22.2	44	61.1	100	94.4	88.9
	Control arm	0/25	48.0	24.0	48.0	68.0	100	92.0	88.0
	Perindopril+VK2 (45 mg)	0/25	48.0	12.0	28.0	32.0	100	100	96.0
	Perindopril	0/19	48.0	15.8	36.8	52.6	100	94.7	89.5
Mizuta et al. [10]	VK2 (45 mg)	1/31	28.9	12.5	39.0	64.3	100	96.6	87.0
	Control arm	3/26	27.7	55.2	83.2	91.6	96.4	80.9	64.0
Kakizaki et al. [11]	VK2 (45 mg)	4/26	-	7.7	51.4	61.2	100	95.0	77.5
	Control arm	7/23	-	28.3	64.1	90.1	95.8	90.2	66.4
Hotta et al. [12]	VK2 (45 mg)	2/19	19.5	23.8	28.6	-	100	100	-
	Control arm	2/22	16.5	33.3	46.5	73.3	87.5	81.7	81.7
Yoshida et al. [13]	VK2 (45 mg)	9/173	36	31.2	-	-	99.2	-	-
	VK2 (90 mg)	5/180	36	37.7	-	-	98.7	-	-
	Control arm	7/174	36	28.2	-	-	97.2	-	-
Kubota et al. [15]	VK2 (45 mg)	50/0	-	20.6	36.9	43.6	-	-	-
	Control arm	51/0	-	30.0	52.0	76.1	-	-	-
Hosho et al. [16]	VK2 (45 mg)	2/21	23.8	26.1	32.8	78.1	100	93.3	85.6
	Control arm	7/42	26.9	49.0	61.7	67.6	93.3	79.1	56.5

Abbreviation: VK2, vitamin K2 analog.

#### Tumor recurrence rate

**Figure 2:**
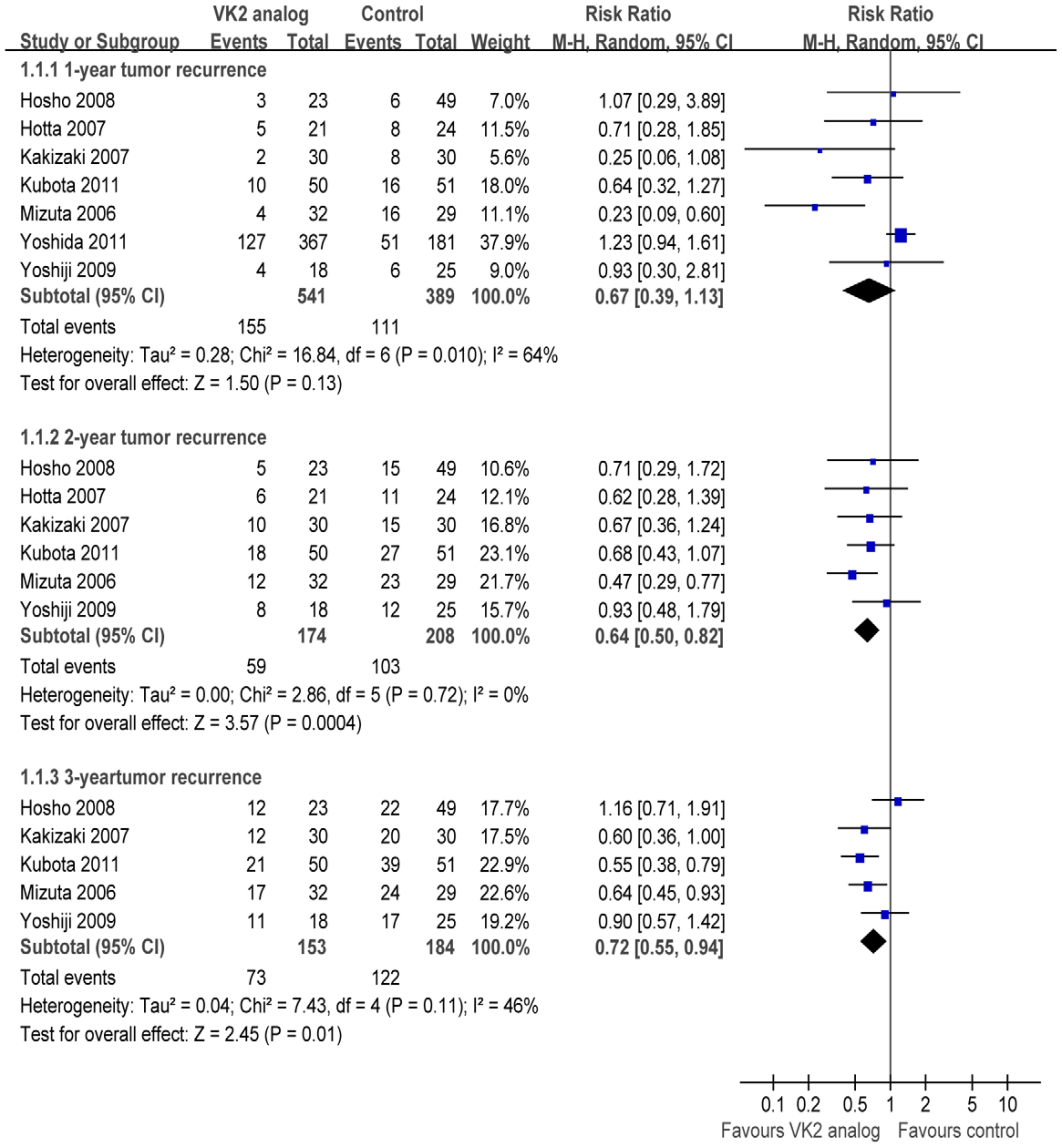
Meta-analysis of tumor recurrence comparing curative treatment plus vitamin K2 analog versus curative treatment alone for patients with hepatocellular carcinoma. CI, confidence interval.

All seven studies [9]–[13], [15]–[16] reported the 1-year recurrence rate. Postoperative VK2 analog therapy did not statistically reduce the incidence of recurrence in patients with HCC after curative treatment; there was statistical heterogeneity (*p* = 0.01, I^2^ = 64%) and the pooled RR based on random-effect model was 0.67 (95% CI 0.39–1.13, *p* = 0.13) (Fig. 2). However, chemopreventive VK2 analog therapy was associated with a significant reduction in the 2- and 3-year recurrence rates. There were statistical heterogeneities (*p* = 0.72, I^2^ = 0%; *p* = 0.11, I^2^ = 46%) and respective pooled RRs based on fixed-effect model were 0.65 (95% CI 0.51–0.83, *p*<0.001) and 0.70 (95% CI 0.58–0.85, *p*<0.001).

Of the 930 patients included in the meta-analysis, 356 (38.3%) received VK2 analog at a dose of 45 mg per day, and 185 (19.9%) at a dose of 90 mg per day. To analyze the possible effect of VK2 analog dose on tumor recurrence, subgroup analysis was performed only on patients who received 45 mg of VK2 analog per day. The 1-year tumor recurrence rate did not differ significantly between the treatment and control arms (RR 0.66, 95% CI 0.41–1.07, *p* = 0.09).

#### OS

**Figure 3:**
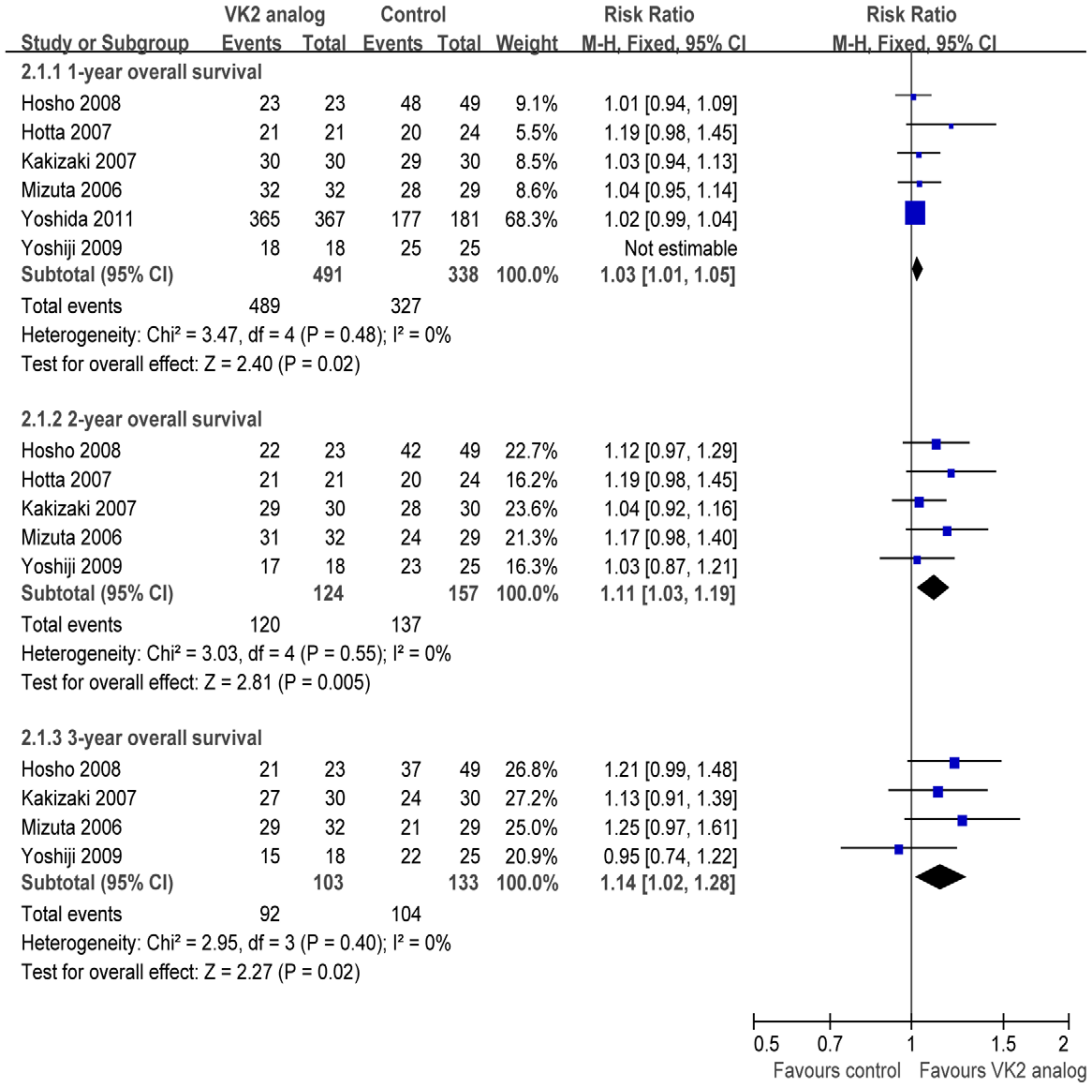
Meta-analysis of overall survival comparing curative treatment plus vitamin K2 analog versus curative treatment alone for patients with hepatocellular carcinoma. CI, confidence interval.

Meta-analysis of 1-year OS was based on seven studies [9]–[13], [15]–[16]. These studies showed a beneficial effect of chemopreventive VK2 analog therapy, with a pooled RR of 1.03 (95% CI 1.01–1.05, *p* = 0.02) without statistical heterogeneity (*p* = 0.48, I^2^ = 0%). Similarly, chemopreventive VK2 analog therapy significantly improved 2- and 3-year OS, with respective pooled RRs of 1.11 (95% CI 1.03–1.19, *p* = 0.005) and 1.14 (95% CI 1.02–1.28, *p* = 0.02) without statistical heterogeneity (*p* = 0.55, I^2^ = 0%; *p* = 0.40, I^2^ = 0%) (Fig. 3).

To analyze the possible effect of VK2 analog dose on OS, subgroup analysis was performed only on patients who received 45 mg of VK2 analog per day. The 1-year OS differed significantly between the treatment and control arm (RR 1.03, 95% CI 1.00–1.06, *p* = 0.02).

### Sensitivity analysis

Excluding the cohort study [16] did not alter the results for 1-, 2- or 3-year recurrence rates. Given that the 1-year tumor recurrence rate showed substantial heterogeneity among the trials, we excluded the trial with maximal weight [13] and obtained a heterogeneity I^2^ of 25% (*p* = 0.24) among the remaining trials. The pooled RR for these trials was 0.56 (95% CI 0.34–0.90, *p* = 0.02).

Similarly, excluding the cohort study [16] did not alter the 1- and 2-year OS. However, it did eliminate the improvement in 3-year OS due to chemopreventive VK2 analog therapy, with a pooled RR of 0.12 (95% CI 0.97–1.28, *p* = 0.12).

### Publication bias

Begg's funnel plots were prepared for the 7 studies [9]–[13], [15]–[16] to assess publication bias for studies about VK2 analog for postoperative HCC. The shape of the funnel plots seemed asymmetrical for the 1-, 2-, 3-year tumor recurrence rate and overall survival, suggesting the presence of publication bias (Figure S1).

### Adverse effects of VK2 analog

None of the seven studies reported any adverse effects, such as abnormal laboratory findings, that were likely to be due to the VK2 analog.

## Discussion

A previous systematic review [8] of postoperative VK2 analog therapy in patients with HCC after curative hepatic resection or local ablation reported that the analog had chemopreventive effects, yet a more recent, much larger-scale RCT [13] did not find such effects. To help resolve the controversy over the benefits of postoperative VK2 analog therapy, we carried out a meta-analysis of all the studies in the previous systematic review and the most recent RCT, which allowed us to maximize the sample size. We examined the effect of VK2 therapy on tumor recurrence and OS. The therapy significantly reduced the 2- and 3-year tumor recurrence rate, though it did not alter the 1-year rate. The therapy also significantly increased 1-, 2- and 3-year OS. The results were similar for VK2 doses of 45 or 90 mg per day. When the meta-analysis was repeated with only RCTs, after excluding one cohort study, similar results were obtained, except for 3-year OS. No significant adverse effects associated with the VK2 analog were reported, suggesting that the therapy is safe.

VK2 is a co-enzyme of γ-carboxylase. Des-γ-carboxy prothrombin has been called Prothrombin Induced by Vitamin K Absence or antagonist-II (PIVKA-II). This abnormal prothrombin is found in elevated concentrations in the serum of patients with HCC. In fact, high preoperative serum PIVKA-II may predict poor prognosis [18]–[20]. Researchers have demonstrated that PIVKA-II stimulates human vascular endothelial cell growth and migration [21], and conversely VK2 may inhibit the growth of HCC cell lines [22], perhaps by inhibiting or activating certain signaling pathways [23]–[26]. Though the precise mechanisms by which VK2 induces cell cycle arrest and growth suppression have not been fully clarified, the rationale of VK2 analog therapy is to prevent a secondary tumor from developing in the liver tissue remaining after curative resection. This chemoprevention approach, designed to halt the appearance of new tumors, differs from adjuvant therapy, which aims to eradicate preexisting microscopic tumor foci that pre-resection imaging modalities fail to detect. Adjuvant therapy cannot prevent HCC recurrence in the long term, making chemoprevention important for achieving long-term tumor-free survival.

The present meta-analysis included seven studies involving patients with HCC lesions treated by curative hepatic resection or local ablation. The proportion of patients treated by radiofrequency ablation of HCC lesions was 84% (780/930). Radiofrequency ablation stimulates the activity of natural killer cells through several mechanisms [27]. Most patients in our meta-analysis presented a single tumor. Mean tumor diameter ranged from 1.1 to 5.0 cm, and most tumors had diameters less than 2 cm (Table 1). In other words, most patients in our study received radical treatment. These patients may have presented with fewer indicators of poor prognosis, such as vascular invasion, tumor multiplicity and large tumor size, all of which are related to early recurrence. Thus, most cases of recurrence in the patients in our meta-analysis would be expected to occur after one year. This may help to explain why VK2 analog therapy was not associated with a reduction in 1-year tumor recurrence in our meta-analysis. Theoretically, patients with HCC that show a lower tumor recurrence rate should also show a higher survival rate. However, since most studies in this meta-analysis had a follow-up of less than 36 months, the long-term preventive efficacy of VK2 analog therapy is unclear from the available evidence.

Our meta-analysis indicates a significant difference in OS between the treatment arm and control arm. Conversely, all seven of the included studies suggested that VK2 analog had no significant effects on OS after hepatic resection or local ablation, and the previous systematic review [8] that included four of these studies concluded that the beneficial effect of VK2 analog on OS was uncertain. Two of the RCTs [10]–[11] included in the present meta-analysis reported that the VK2 analog reduced HCC recurrence; for example, Mizuta and coworkers [10] reported a hazard ratio for HCC recurrence following VK2 analog therapy of 0.27 (95% CI 0.12–0.60, *p* = 0.001). However, Yoshida and coworkers [13] found no difference in disease-free survival between treatment and control arms (hazard ratio, 1.15, 95% CI 0.84–1.57, one-sided, *p* = 0.811). Subgroup analysis based on type of recurrence and certain tumor-related factors also produced negative results [13]. The discrepancies between individual studies and meta-analyses on this topic highlight the importance of using as large a sample as possible.

All the studies in this meta-analysis were conducted in Japan. Most patients (83%) were also infected with hepatitis C virus. Therefore, the efficacy of VK2 analog therapy in other patient populations or in patients infected with hepatitis B virus is unknown. Nevertheless, studies conducted in China and Japan suggest that the VK2 analog may prevent formation of a secondary tumor in residual liver tissue by inhibiting or activating certain signaling pathways [23]–[25]. Therefore, trials of VK2 analog therapy conducted outside Japan may give results similar to those in the present meta-analysis.

This meta-analysis has a significant limitation in that data for 548 (59%) of the 930 patients came from the trial by Yoshida and coworkers [13]. This trial was discontinued at the second interim analysis because investigators found that VK2 did not prevent short-term recurrence [13]. Thus, survival data for most of the patients in our meta-analysis are limited to one year. The long-term efficacy of VK2 analog needs to be validated by more RCTs. In addition, almost no included studies particularly reported the treatment modality after recurrence. The beneficial effects of VK2 analog are shown in later period. Treatment of HCC after recurrence might affect the clinical prognosis.

This meta-analysis has other limitations, due primarily to limitations of the included studies. In Hotta *et al.* [12], the baseline level of serum des-γ-carboxy prothrombin was significantly different between the two arms. Not all studies included here described the method of randomization or allocation concealment. As a result, risk of selection bias and performance bias cannot be excluded. Only one study [13] reported the sample size calculation, and only two [10], [13] used the intention-to-treat principle. Therefore, the results and conclusions of this meta-analysis should be interpreted with caution. Future clinical trials should strive to avoid these limitations as much as possible.

In conclusion, our meta-analysis suggests that VK2 analog therapy shows some benefit in reducing the recurrence rate and increasing OS in patients with HCC after hepatic resection or local ablation. In addition, the oral VK2 analog appears to be safe. However, our results should be interpreted with caution because only 1-year survival data were available for 59% of the patients in the meta-analysis. Future studies should analyze the possible benefits of combining two or more postoperative therapies.

This meta-analysis is guided by the PRISMA statement (Checklist S1).

## Supporting Information

Checklist S1 **PRISMA 2009 Checklist.**
(DOC)

Figure S1 **Begg's funnel plots to examine publication bias for studies about vitamin K2 analog for postoperative hepatocellular carcinoma.**
(TIF)
