# Supplementary figures and images for: Postoperative Use of the Chemopreventive Vitamin K2 Analog in Patients with Hepatocellular Carcinoma
Source: PLoS One. 2013 Mar 7;8(3):e58082. doi: 10.1371/journal.pone.0058082 (PMC3591458; doi:10.1371/journal.pone.0058082)

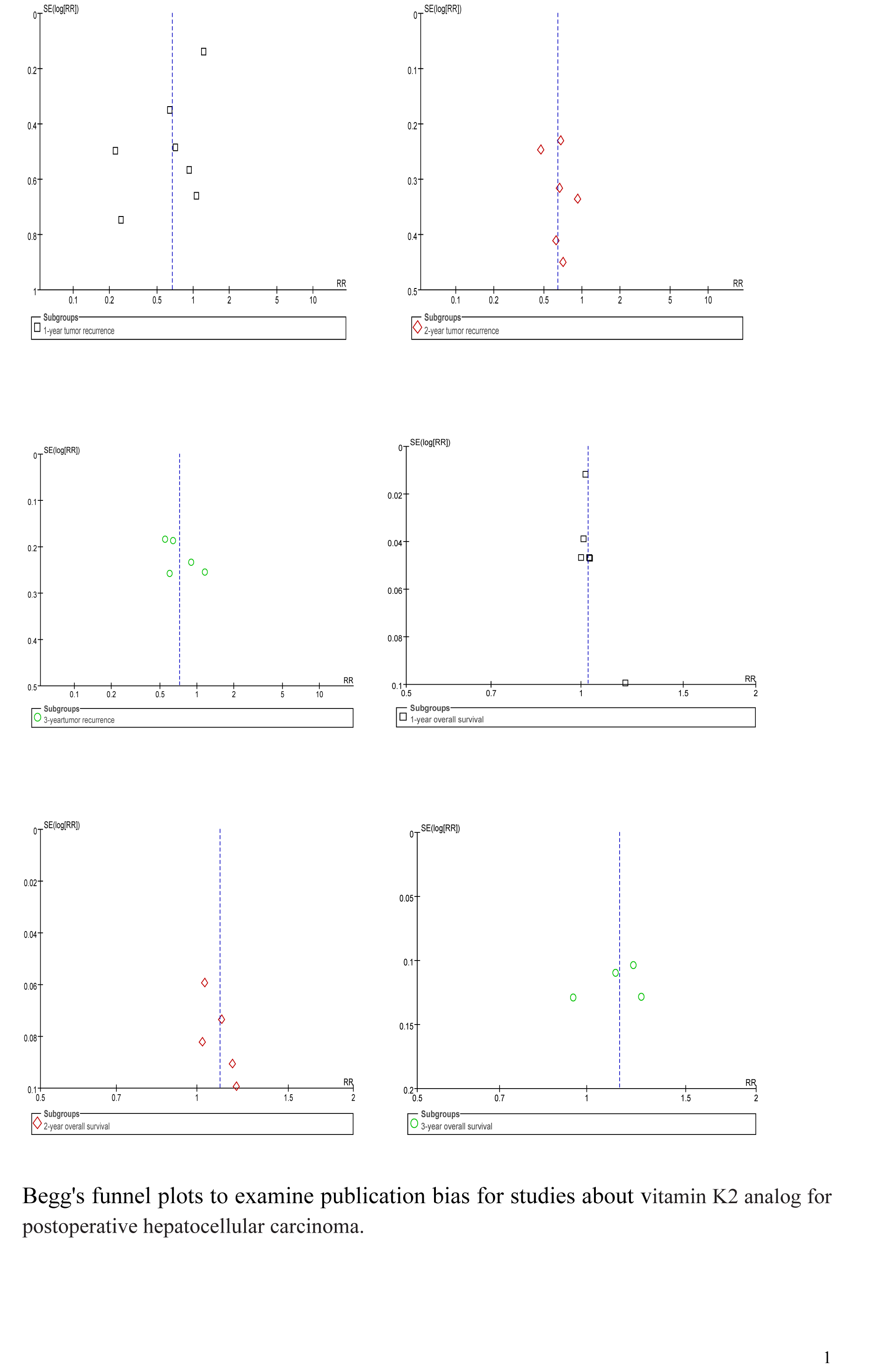

Supplement: Figure S1 — Begg's funnel plots to examine publication bias for studies about vitamin K2 analog for postoperative hepatocellular carcinoma. (TIF) [file pone.0058082.s002.tif]
